# Urban-rural differences in healthcare utilization among beneficiaries in China’s new cooperative medical scheme

**DOI:** 10.1186/s12889-021-11573-3

**Published:** 2021-08-06

**Authors:** Dian Luo, Jing Deng, Edmund R. Becker

**Affiliations:** 1 Department of Population Health Sciences, School of Medicine and Public Health, University of Wisconsin, Madison, WI USA; 2 School of Public Health and Management, Chongqing Medical University, Chongqing, China; 3 The Research Center for Medicine and Social Development, The Collaborative Innovation Center for Social Risk Governance in Health, Chongqing, China; 4 Department of Health Policy and Management, Rollins School of Public Health at Emory University, 1518 Clifton Road NE, 30322 Atlanta, GA USA

**Keywords:** Healthcare utilization, Healthcare expenditure, New cooperative medical scheme, China, CHARLS 2015.

## Abstract

**Background:**

The New Cooperative Medical Scheme (NCMS) is a voluntary social health insurance program launched in 2002 for rural Chinese residents where 80% of people were without health insurance of any kind. Over time, several concerns about this program have been raised related to healthcare utilization disparities for NCMS participants in urban versus rural regions. Our study uses 2015 national survey data to evaluate the extent of these urban and rural disparities among NCMS beneficiaries.

**Methods:**

Data for our study are based on the Chinese Health and Retirement Longitudinal Study (CHARLS) for 2015. Our 12,190-patient sample are urban and rural patients insured by NCMS. We use logistic regression analyses to compare the extent of disparities for urban and rural residence of NCMS beneficiaries in (1) whether individuals received any inpatient or outpatient care during 2015 and (2) for those individuals that did receive care, the extent of the variation in the number of inpatient and outpatient visits among each group.

**Results:**

Our regression results reveal that for urban and rural NCMS patients in 2015, there were no significant differences in inpatient or outpatient utilization for either of the dependent variables – 1) whether or not the patient had a visit during the last year, or 2) for those that had a visit, the number of visits they had. Patient characteristics: age, sex, employment, health status, chronic conditions, and per capita annual expenditures – all had significant impacts on whether or not there was an inpatient or outpatient visit but less influence on the number of inpatient or outpatient visits.

**Conclusions:**

For both access to inpatient and outpatient facilities and the level of utilization of these facilities, our results reveal that both urban and rural NCMS patients have similar levels of resource utilization. These results from 2015 indicate that utilization angst about urban and rural disparities in NCMS patients do not appear to be a significant concern.

## Background

The New Cooperative Medical Scheme (NCMS) in China, established in 2002 to provide financial protection for rural residents, was a voluntary program designed to facilitate cost sharing between government and rural residents. The NCMS is designed to reduce the financial burden of illness on the rural population focusing mostly on inpatient services and some outpatient services [1]. Beneficiaries pay a flat-rate premium in return for a uniform benefit package. As a result, beneficiaries have the opportunity to get greater access to healthcare utilization with corresponding increases in reimbursement and overall healthcare expenditures. The overall responsibility for managing and supervising the NCMS falls under the auspices of the Ministry of Health which has the overall responsibility to manage and supervise the plan. The specific policy implementation responsibilities of the plan are decentralized to county level governments. By the end of 2009, calculations show that 95.3% of all counties and 91.5% (815 million) of all rural population were covered by the NCMS [2].

The New Cooperative Medical Scheme (NCMS) in China has raised many concerns and the potential extent of the disparities in healthcare utilization and expenditures which are critical concerns to the application and improvement of the program [3–6]. The focus of the disparity concerns focus on issues related to the extent of NCMS benefits that are alleged to be concentrated in small segments of the populations that are economically affluent instead of being distributed equally. Research has shown that more economically affluent groups are more likely to consume higher levels of medical services and proportionately account for greater levels of reimbursement and expenditures [7–9]. However, since NCMS is a Social Medical Insurance (SMI) program and designed to be a financing plan for mobilizing funds and pooling health risks, the original intent of the program was to marshal healthcare funds for the poor and near-poor rural residents not the economically affluent participants [10].

Initially, when the NCMS was established in 2002, rural residents typically clustered in rural areas with similar economic profiles and disparities in healthcare utilization and expenditures were not a major concern among beneficiaries. However, with economic prosperity and rapidly growing urbanization, an increasing number of rural residents migrated from rural to urban areas to seek better employment opportunities. This rural migration had grown rapidly and by 2015 was estimated to have involved over 275 million Chinese residents [11]. Consequently, these rural migrants play a significant role in the current Chinese SMI program and impact a large share of the rural population.

However, when compared with the original urban residents, these rural migrants have had limited access to a range of social services due to the Chinese unique household registration system (hukou system), which includes insurance coverage for healthcare services [12]. The hukou is a family registration program for all households and serves as a domestic passport, regulating population distribution and rural-to-urban migration. Individuals born in rural areas have been assigned to a rural hukou status while individuals born in urban areas are assigned to urban hukou. Once assigned, a hukou status is hard to change and typically stays unchanged even when an individual moves from a rural to urban area [13]. Consequently, there are large numbers of rural migrants who live in urban areas with a rural hukou status, are insured by NCMS, and who represent economically affluent beneficiaries.

We ask the question - Do these rural migrants who are more affluent but still insured by NCMS get a disproportionate share of healthcare services? That is, do these recent rural migrants create disparities in healthcare utilization and expenditures due to their affluence when compared to other those still residing in a rural region? We might hypothesize that their higher economic profiles enable them to have access to more healthcare services and thus get greater reimbursement. As a result, this unequal distribution of benefits may lead to an inefficient use of risk pooling and thus undermine an original goal of NCMS [14]. However, in contrast, we would also note that many rural migrants are also constrained by major barriers in seeking care including: (1) limited knowledge about NCMS benefits, (2) difficult dealing with new and different reimbursement policies (destination bill policy), and (3) a general lack of good health practices and awareness [15–18].

Given these criticisms in the NCMS, it is unclear the extent of the disparities among NCMS beneficiaries. Furthermore, there has been a dearth of research studies analyzing the disparities in healthcare utilization of those NCMS patients with a rural hukou residing in an urban area (migrants) versus those NCMS patients with rural hukou status but still living in rural regions. Our study seeks to fill this research gap by evaluating the extent of the disparities in healthcare utilization levels between NCMS beneficiaries in urban and rural Chinese communities.

## Methods

### Database

The data for this investigation are taken from the 2015 Chinese and Health and Longitudinal Retirement Survey (CHARLS). The CHARLS is a nationwide representative survey that captures the social, financial, and health characteristics of the mainland China population. The survey instruments capture detailed information on participant’s: background, family structure, financial support, health condition, functional status, insurance coverage, employment status, scope of retirement and pension benefits, income level, annual expenses and assets, and housing conditions. Using a probability proportional to size (PPS) sampling strategy, the CHARLS survey includes 450 communities in 150 counties from 28 of the 32 provinces in mainland China. The randomly selected households surveyed are gathered from within each rural or urban community. Surveyed residents had to be 45 years or older and, with their spouses included, were initially interviewed at baseline and again after 2-years.

There were four major steps used to create the CHARLS dataset. First, county-level areas (counties or urban districts) were directly sampled. The scope of counties surveyed covered 28 of 32 provinces in mainland China but excluded Tibet. The second step used actual village-level population data from the National Bureau of Statistics to refine the selection of villages and community units within the county units. Using this data, primary sampling units (PSUs) were then created for 450 PSUs and using a probability proportional-to-size (PPS) sampling methodology, three PSUs were selected in each county-level unit. In the third step, household units were chosen from each PSU based on a sampling structures constructed by Google Earth based maps. In the final step, all respondents were required to fill out the modules in the survey instrument, noted above, through a personal interview program that was organized and facilitated by a laptop [19].

### Sample

**Fig. 1:**
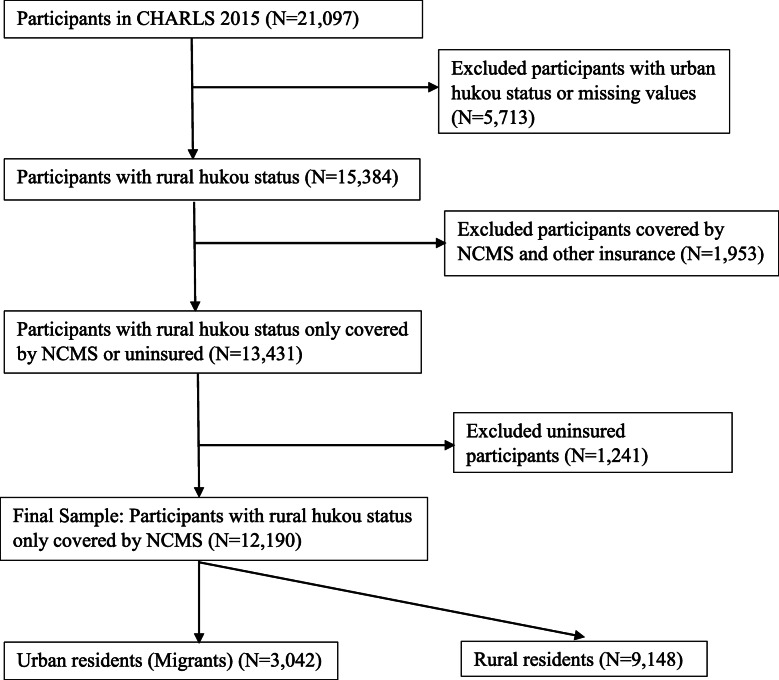
Flow Chart for Study Participants from the 2015 CHARLS Survey

As shown in Fig. 1, a total of 21,097 individuals from 12,235 households participated in the2015 CHARLS survey. Participants in an urban hukou or with a missing value for hukou status were dropped. Additionally, there were 1953 participants that had either missing data or had insurance coverage in addition to NCMS coverage and these participants were also dropped. Finally, we dropped 1241 participants who were not covered by any health insurances. The final sample contained 12,190 participants with just rural hukou status. Among these 12,190 NCMS beneficiaries with the rural hukou status, 3042 or 25% lived in an urban area in 2015 while 9148 or 75% of the sample lived in a rural area.

### Dependent variables

We constructed four dependent variables. Two of the variables were an inpatient and outpatient dummy variable constructed for whether the individual had any healthcare visit in either setting in 2015. The other two variables were constructed based on the number of inpatient and outpatient visits for each participant during the month. That is, for those participants that had an inpatient or outpatient visit in 2015, how many visits did they have in the most recent month in either the inpatient or outpatient setting.

### Independent variables

To develop our conceptual framework, we used an updated Anderson Health Services Utilization Model as our starting point to select independent variables. We include several additional independent variables identified by Anderson and colleagues in later models to more fully understand patient utilization and to more completely explain our outcomes. We included total annual household consumption per capita instead of total annual household income per capita to measure living standard [20]. Since many participants in our rural hukou are elderly, retired, and/or working in agricultural areas with varying incomes in different growing seasons, we felt total household consumption was a better variable to measure their overall financial condition. In addition, given the elderly nature of the CHARLS survey and the low numbers of young people in the sample, we combined all people younger than 45 years of age into a single category for the age variable.

The resulting list of patient characteristics in our study capture critical elements in the utilization of health care services and conceptually frame three aspects of a participant’s health status: 1) predisposing characteristics: sex, patient age, household living conditions, marital status, and level of educational attainment; 2) enabling resources: total household annual expenditure per capita (PCE) and participant’s work status; 3) health needs: self-report health status, range of chronic disease condition, and body mass index (BMI) [14]. Table 1 reports the relevant descriptive characteristics and statistical significant levels for all these variables.

**Table 1:** Sample Size and Demographic Characteristics of Population with NCMS Urban Designation in the 2015 CHARLS Survey

	**Overall**		**Urban**		**Rural**		***p*-value**
**Sample Size**	**N**	**%**	**N**	**%**	**N**	**%**	
**CHARLS Survey of Urban NCMS Sample**	**12,190**	**100**	**3042**	**24.9**	**9148**	**75.1**	
**Dependent Variables**							
**Inpatient Visit in 2015 (0,1)**	**12,178**	**100**	**3040**	**25.0**	**9138**	**75.0**	
**Number of Individuals with at Least One Inpatient Visit in Last Year**	**1612**	**13.3**	**407**	**13.4**	**1210**	**13.2**	**0.84**
**Number of Individuals without Inpatient Visit in Last Year**	**10,561**	**86.7**	**2633**	**86.6**	**7928**	**86.8**	
**Outpatient Visit in 2015 (0,1)**	**12,042**	**100**	**2988**	**24.8**	**9054**	**75.2**	
**Number of Individuals with at Least One Outpatient Visit in Last Month**	**2263**	**18.8**	**544**	**18.2**	**1719**	**19.0**	**0.34**
**Number of Individuals without Inpatient Visit in Last Year**	**9779**	**81.2**	**2444**	**81.8**	**7335**	**81.0**	
**Demographic Characteristics**	**Overall**		**Urban**		**Rural**		***p*-value**
**Dependent Variables**	**Mean**	**SD**	**Mean**	**SD**	**Mean**	**SD**	
**Number of Inpatient Visits in Last Year for those with at least one inpatient visit**	**1.45**	**0.99**	**1.39**	**0.92**	**1.47**	**1.02**	**0.17**
**Number of Outpatient Visits in Last Month for those with at least one outpatient visit**	**2.23**	**2.40**	**2.16**	**2.27**	**2.25**	**2.44**	**0.42**
**Independent Variables**	**N**	**%**	**N**	**%**	**N**	**%**	***p*-value**
**Age**							
**< 45**	**713**	**5.85**	**186**	**6.13**	**527**	**5.76**	**< 0.01**
**45–54**	**3752**	**30.8**	**989**	**32.58**	**2763**	**30.22**	
**55–64**	**3843**	**31.55**	**983**	**32.38**	**2860**	**31.28**	
**65+**	**3872**	**31.79**	**878**	**28.92**	**2994**	**32.74**	
**Sex**							
**Male**	**5655**	**46.39**	**1348**	**44.31**	**4307**	**47.08**	**< 0.01**
**Female**	**6535**	**53.61**	**1694**	**55.69**	**4841**	**52.92**	
**Education**							
**No formal education**	**3599**	**29.57**	**706**	**23.25**	**2893**	**31.68**	**< 0.01**
**< =Elementary school**	**5850**	**48.07**	**1583**	**52.12**	**4267**	**46.72**	
**> =Middle school**	**2721**	**22.36**	**748**	**24.63**	**1973**	**21.6**	
**Marital status**							
**Living with spouse**	**9785**	**80.27**	**2487**	**81.76**	**7298**	**79.78**	**< 0.05**
**Living without spouse**	**2405**	**19.73**	**555**	**18.24**	**1850**	**20.22**	
**Work status**							
**No employment**	**3018**	**25.05**	**922**	**30.85**	**2096**	**23.13**	**< 0.01**
**Agricultural work**	**6088**	**50.52**	**1066**	**35.66**	**5022**	**55.42**	
**Non-agricultural work**	**2944**	**24.43**	**1001**	**33.49**	**1943**	**21.44**	
**Self-reported health status**							
**Good**	**1866**	**16.16**	**491**	**17.2**	**1375**	**15.82**	**< 0.01**
**Fair**	**6111**	**52.93**	**1605**	**56.24**	**4506**	**51.84**	
**Poor**	**3569**	**30.91**	**758**	**26.56**	**2811**	**32.34**	
**Chronic disease status**							
**No chronic disease**	**3010**	**30.18**	**772**	**30.93**	**2238**	**29.92**	**< 0.01**
**Diabetes**	**1428**	**14.32**	**347**	**13.9**	**1081**	**14.45**	
**Hypertension**	**1686**	**16.9**	**456**	**18.27**	**1230**	**16.45**	
**Diabetes & Hypertension**	**589**	**5.9**	**178**	**7.13**	**411**	**5.5**	
**Other**	**3262**	**32.7**	**743**	**29.77**	**2519**	**33.68**	
**BMI**							
**Underweight (< 18.5)**	**636**	**5.25**	**127**	**4.21**	**509**	**5.6**	**< 0.01**
**Healthy (18.5–25)**	**5857**	**48.39**	**1306**	**43.32**	**4551**	**50.07**	
**Overweight (25–30)**	**2815**	**23.26**	**790**	**26.2**	**2025**	**22.28**	
**Obese (> 30)**	**2796**	**23.1**	**792**	**26.27**	**2004**	**22.05**	
**Per Capita Annual Expenditure (PCE)**							
**Bottom (<6420rmb)**	**3212**	**39.42**	**688**	**34.71**	**2524**	**40.93**	**< 0.01**
**Middle (6420-13187rmb)**	**2707**	**33.22**	**723**	**36.48**	**1984**	**32.17**	
**Top (>13187rmb)**	**2230**	**27.37**	**571**	**28.81**	**1659**	**26.9**	

(Not all counts sum to 12,190 due to missing values for some of the independent variables)

### Statistical analysis

We use descriptive and regression analyses to compare these two NCMS populations. We used chi-square test in our descriptive Table 1 to examine urban-rural differences among different demographic characteristics. We use logistic regression to examine two of our four dependent variables: inpatient and outpatient dummy variables were constructed to indicate whether the individual had any healthcare visit in either setting in 2015. The other two dependent variables - the number of inpatient or outpatient monthly visits for those that had at least one visits in 2015 – were analyzed with an ordinary least squares (OLS) regression model.

In order to increase the statistical power, we create dummy variable in each independent variable. The level value was coded as 1, and all other values including missing values were coded as 0. All our analyses are conducted with StataSE 16 (StataCorp, College Station, USA).

## Results

### Demographic characteristics

Among NCMS beneficiaries, Table 1 indicates that 13.3% of urban and rural NCMS beneficiaries had an inpatient visit in the past year with urban NCMS beneficiaries being slightly more likely to have an inpatient visit, 13.4%, than a rural NCMS beneficiary, 13.2%. These differences were not statistically significant (*p* = 0.84). For the number of annually inpatient visits, urban NCMS beneficiaries have average slightly fewer inpatient visits, 1.39, versus, 1.47, inpatient visits for rural NCMS beneficiaries. The *p*-value of 0.17 reflected that these urban-rural difference for NCMS beneficiaries in outpatient visits were not statistically significant.

Table 1 also indicates that 18.8% of urban and rural NCMS beneficiaries had an outpatient visit in the past month with urban NCMS beneficiaries being slightly less likely to have an outpatient visit, 18.2%, than a rural NCMS beneficiary, 19.0%. These differences were not statistically significant (*p* = 0.34). For the number of monthly outpatient visits, urban NCMS beneficiaries have average slightly less outpatient visits, 2.27, versus, 2.44, outpatient visits for rural NCMS beneficiaries. The *p*-value of 0.42 reflected that these urban-rural difference for NCMS beneficiaries in outpatient visits were not statistically significant.

In contrast, all the urban/rural demographic characteristics for NCMS beneficiaries are significant at the < 0.05 or greater. Compared to rural NCMS beneficiaries, urban NCMS beneficiaries, those that migrated from rural to urban areas, typically are a little older, have a higher educational level, higher self-reported health status, lower BMI, and are more likely to be unemployed. Specifically, when we compare age groupings, we find urban NCMS beneficiaries are less concentrated in over 65 years old age group compared to rural NCMS beneficiaries: 28.92% versus 32.74%. Urban NCMS beneficiaries have a higher proportion of females compared to rural NCMS beneficiaries: 55.69% versus 52.92%.

Educational levels for urban NCMS beneficiaries with rural hukou status have a significantly higher level of education than rural NCMS beneficiaries. Just 23.25% of urban NCMS beneficiaries lack formal education while 31.68% of rural beneficiaries lack formal education (*p* < 0.01). Additionally, urban NCMS beneficiaries are slightly more likely to be married (81.76% vs. 79.78%, *p* < 0.01). Not surprisingly, urban NCMS beneficiaries are less likely to be engaged in agricultural work compared to rural NCMS beneficiaries (35.66% vs. 55.42%, *p* < 0.01). However, in contrast, urban NCMS beneficiaries are more likely to be unemployed than rural NCMS beneficiaries (30.85% vs. 23.13%, *p* < 0.01).

In terms of their health status, urban beneficiaries report ‘good health’ slightly more than rural NCMS beneficiaries (17.20% vs. 15.82%, p < 0.01) and, correspondingly, the overall absence of chronic diseases is similar in both urban and rural NCMS populations (30.93% vs. 29.92%, p < 0.01). Hypertension and/or diabetes are the most prevalent and severe diseases among both subgroups with over 35% of participants in both urban and rural areas covered by NCMS with these chronic conditions. BMI shows that nearly 50% of the total sample size are identified as overweight or obese. Compared to rural NCMS beneficiaries, urban NCMS beneficiaries have a slightly higher proportion of both overweight (26.20% vs. 22.28%) and obesity groups (26.27% vs. 22.05%). Urban NCMS beneficiaries have significantly higher annual household consumption per capita than rural NCMS beneficiaries with over 6% more NCMS beneficiaries in the middle and top annual levels of household consumption.

### Logistic regression results

#### Inpatient care visits during the last year

The logistic regression results are reported in Table 2 for the dependent variables inpatient care visit during the past year and outpatient care visit during the past year, respectively. For an inpatient care visit during the past year, there is no significant difference between urban and rural NCMS beneficiaries indicating that, for these two groups, they have similar likelihood of having an inpatient care visit (OR = 0.933,*p* > 0.1). This would indicate that NCMS migrants that have moved from rural to urban areas in China appear to have similar access to inpatient care to those NCMS beneficiaries that remained in rural areas.

**Table 2:** Logistic Regression Results for Having Any Inpatient Care/Outpatient Care Visit in the Last Year/Last Month

Had a visit last year/last month (no = 0, yes = 1)	Inpatient care (*N* = 12,178)			Outpatient care (*N* = 12,042)		
**Insurance Type (Ref = Rural covered)**	**OR**	**CI**		**OR**	**CI**	
**Urban covered**	0.933	(0.821,	1.061)	0.996	(0.891,	1.114)
**Age (Ref = < 45)**						
**45+**	1.435**	(1.037,	1.986)	1.271**	(1.006,	1.607)
**55+**	1.600***	(1.154,	2.217)	1.156	(0.910,	1.469)
**65+**	2.035***	(1.465,	2.828)	1.326**	(1.038,	1.693)
**Sex (Ref = female)**						
**Male**	0.864**	(0.765,	0.976)	1.400***	(1.260,	1.554)
**Education level (Ref = No formal)**						
**Elementary**	0.929	(0.814,	1.061)	1.092	(0.973,	1.226)
**Middle**	0.899	(0.756,	1.069)	1.072	(0.924,	1.244)
**Marital Status (Ref = Unmarried)**						
**Married**	0.979	(0.854,	1.122)	1.016	(0.902,	1.144)
**Job (Ref = No employment)**						
**Agricultural**	0.681***	(0.599,	0.774)	1.095	(0.974,	1.232)
**Nonagricultural**	0.548***	(0.457,	0.657)	1.016	(0.872,	1.184)
**Health Status (Ref = Poor)**						
**Good**	0.210***	(0.167,	0.263)	0.214***	(0.176,	0.259)
**Fair**	0.350***	(0.311,	0.394)	0.484***	(0.438,	0.536)
**Chronic Diseases (Ref = No Diseases)**						
**Diabetes**	1.338***	(1.120,	1.598)	1.294***	(1.109,	1.509)
**Hypertension**	1.201**	(1.006,	1.433)	1.153*	(0.991,	1.342)
**Diabetes & Hypertension**	1.683***	(1.337,	2.118)	1.222*	(0.981,	1.523)
**Other**	1.343***	(1.166,	1.547)	1.196***	(1.061,	1.348)
**BMI (Ref = Normal)**						
**Healthy**	0.998	(0.801,	1.243)	0.953	(0.786,	1.155)
**Overweight**	1.203	(0.949,	1.525)	0.917	(0.744,	1.129)
**Obese**	1.152	(0.911,	1.456)	0.854	(0.694,	1.052)
**Per Capita Annual Expenditure (Ref = Bottom)**						
**Middle**	1.193**	(1.037,	1.373)	1.083	(0.962,	1.219)
**Top**	1.671***	(1.451,	1.924)	1.161**	(1.023,	1.317)

We show odds ratios here, and 95% confidence interval in parentheses. Counts do not sum to 12,190 due to missing values for some of the independent variables

***Significant at 1% level (two-tailed test)

**Significant at 5% level (two-tailed test)

*Significant at 10% level (two-tailed test)

Reviewing other demographic characteristics, we find their impacts and significant levels consistent with expectations. Not surprisingly, age is significantly related to an inpatient care visit with the elderly being more likely to seek inpatient care with the odds ratios getting progressively larger for each age group when compared to the excluded category less than 44 years of age (age 45+: OR = 1.435, *p* < 0.001; age 55+: OR = 1.600, *p* < 0.001; age 65+: OR = 2.035, *p* < 0.001).

Males are about 14% less likely to have had an inpatient visit in the past year (OR = 0.864, *p* < 0.05) while NCMS beneficiaries that were employed in agricultural work or nonagricultural work experienced 12 and 45%, respectively, less likelihood of having an inpatient visit during the year (agricultural workers: OR = 0.681, *p* < 0.001; nonagricultural workers: OR = 0.548, *p* < 0.001).

As might be expected, health care status and chronic conditions were both strong predictor of an NCMS inpatient visit during 2015. Compared to the self-reported status of poor health, surveyed patients reporting ‘good’ health status had the lowest likelihood of an inpatient visit while those surveyed reporting ‘fair’ health status reported significantly lower likelihood of a visit but nearly double the likelihood of those report ‘good’ health status (Good: OR = 0.210, *p* < 0.001; Fair: OR = 0.350, p < 0.001).

Compared to NCMS beneficiaries without any chronic conditions, any chronic disease was significantly associated with an inpatient visit. NCMS beneficiaries who had both diabetes and hypertensions had the highest possibility of having inpatient visit during the year (OR = 1.683, *p* < 0.001) although having just diabetes (OR = 1.338, *p* < 0.001) or hypertension (OR = 1.201, *p* < 0.05) also resulted in a significant higher likelihood of an inpatient visits.

PCE was also significantly related to having an inpatient care visit. NCMS individuals with higher PCE were associated with a higher likelihood of inpatient care (Middle: OR = 1.193, *p* < 0.05; Top: OR = 1.671, *p* < 0.001). The odds ratio for the top PCE suggests that these NCMS individuals with these levels of household income are 67% more likely to have had an inpatient visit.

Of note, the other three variables - education level, marital status, and BMI were not significantly associated with the likelihood of an inpatient visit.

#### Outpatient care visits during the year

Our logistic regression results, for the dependent variable, the likelihood of an outpatient visit during the previous year for the urban and rural NCMS patient, shown in Table 3, parallel the inpatient care visit logistic regression results. Compared to rural NCMS individuals, the likelihood of an outpatient visit during the past year is not significantly different from the likelihood of a visit by urban NCMS patients (OR = 0.996, *p* = 0.950). There appears to be no difference in the rural and urban NCMS patients in their likelihood of having an inpatient hospital visit.

**Table 3:** Multiple Linear Regression Results for the Number of Inpatient Care/Outpatient Care Visit in the Last Year/Last Month

Visit Frequency (Only including visit > =1)	Inpatient care (*N* = 1612)		Outpatient care (*N* = 2259)	
Insurance Type (Ref = Rural covered)	B	SE	B	SE
**Urban covered**	0.069	(0.057)	0.046	(0.120)
**Age (Ref = < 45)**				
**45+**	−0.037	(0.152)	−0.210	(0.258)
**55+**	0.058	(0.153)	−0.109	(0.263)
**65+**	0.076	(0.152)	−0.107	(0.268)
**Sex (Ref = female)**				
**Male**	−0.019	(0.055)	0.078	(0.113)
**Education level (Ref = No formal)**				
**Elementary**	0.012	(0.059)	−0.204*	(0.123)
**Middle**	−0.015	(0.077)	−0.388**	(0.162)
**Marital Status (Ref = Unmarried)**				
**Married**	−0.108*	(0.061)	−0.172	(0.127)
**Job (Ref = No employment)**				
**Agricultural**	−0.229***	(0.057)	0.147	(0.125)
**Nonagricultural**	−0.244***	(0.082)	0.080	(0.167)
**Health Status (Ref = Poor)**				
**Good**	−0.352***	(0.108)	−0.825***	(0.220)
**Fair**	−0.258***	(0.054)	−0.416***	(0.107)
**Chronic Diseases (Ref = No Diseases)**				
**Diabetes**	0.002	(0.079)	0.213	(0.166)
**Hypertension**	0.071	(0.079)	−0.048	(0.162)
**Diabetes & Hypertension**	−0.039	(0.099)	0.022	(0.235)
**Other**	0.089	(0.064)	0.218*	(0.127)
**BMI (Ref = Normal)**				
**Healthy**	0.055	(0.097)	0.302	(0.201)
**Overweight**	0.057	(0.105)	0.262	(0.218)
**Obese**	0.120	(0.103)	0.262	(0.218)
**Per Capita Annual Expenditure (Ref = Bottom)**				
**Middle**	−0.034	(0.063)	0.121	(0.128)
**Top**	0.104*	(0.061)	−0.170	(0.134)

We show coefficient here, and standard errors in parentheses

***Significant at 1% level (two-tailed test)

**Significant at 5% level (two-tailed test)

*Significant at 10% level (two-tailed test)

Other demographic characteristics were significant to varying degrees. Age again was significant for all age groups except the 55–64 age group with the 65 or older age group having greatest impact. (45+: OR = 1.271, *p* < 0.05; 55+: OR = 1.156, *p* > 0.1; 65+: OR = 1.326, *p* < 0.05). Interestingly, in contrast to the hospital visit regressions in Table 2 for the inpatient visits, males were significantly more likely, about 40%, to have had an outpatient visit than females (OR = 1.400, *p* < 0.001). Self-reported health status again was highly related to the likelihood of an inpatient care visit. Compared to the excluded health status - ‘poor’ health, an NCMS patient reporting good health status had significantly fewer outpatients (Good: OR = 0.214, *p* < 0.01; Fair: OR = 0.484, *p* < 0.01).

Chronic diseases showed a mixed pattern of significance with an outpatient visit with only diabetes and the ‘other’ category showing a statistically significant influence. Diabetes had the highest influence on an NCMS beneficiary seeking outpatient care (OR = 1.294, *p* < 0.001) while for ‘other’ chronic conditions the impact was slightly smaller (OR = 1.196, *p* < 0.01).

Among the CPE categories, only the highest CPE was significantly related to having an outpatient care visit (Top: OR = 1.161, *p* < 0.05).

In this regression, four variables showed no significant relationship to the likelihood of having an outpatient visit: education level, marital status, BMI, and employment status with the first three variables also being insignificant in both the NCMS inpatient and outpatient logistic regressions.

### OLS regression results

#### Number of annually visits for inpatient care

The OLS regression results are shown in Tables 3. These inpatient and outpatient monthly visit results use the same set of independent variables as Tables 2 and 3 but the dependent variables are the number of monthly inpatient or outpatient care visits, respectively, for NCMS patients and only includes patients that had at least one visit.

When compared to rural NCMS individuals, the number of monthly visits for urban NCMS inpatients again shows no statistically significant difference for this population when compared to rural NCMS inpatients (Coef = 0.069, *p* > 0.10).

For the other independent variables, only two - employment status and health status – showed statistical significance. Unemployed NCMS individuals had more monthly inpatient visits than agricultural or nonagricultural workers (agricultural: Coef = − 0.229, *p* < 0.001; nonagricultural: Coef = − 0.244, *p* < 0.05). Similarly, self-report health status was significantly related to the number of monthly inpatient visits. Compared to those NCMS patients with ‘poor’ health status’, patients with ‘fair’ and ‘good’ health status utilized significantly fewer inpatient visits (Good: Coef = − 0.352p < 0.001; Fair: Coef = − 0.258, *p* < 0.001).

Among other independent variables, only two variables - married individuals and the ‘Top’ PCE - approached statistical significance. For married individuals, the coefficient is negative, suggesting they tended to have lower number of inpatient visits than non-married individuals (Coef = − 0.108, *p* < 0.1). For the independent variable ‘Top’ PCE, the coefficient was positive, indicating potentially higher visit utilization but, again, not significant at the 0.05 level (Coef = 0.104, *p* < 0.1).

Surprisingly, another independent variable with no statistical significance was chronic conditions. When compared to beneficiaries without chronic conditions, none of the chronic diseases were significantly associated with a greater number of NCMS monthly inpatient visits. In addition, five other independent variables in the number of monthly inpatient visits regression showed any significant results: age, sex, education level, chronic conditions, and BMI.

#### Number of monthly visits for outpatient care

For outpatient care, with the number of monthly outpatient visits as the dependent variable, our OLS regressions again showed similar results with those for inpatient care. When compared to rural NCMS individuals, the number of monthly outpatient visits for urban NCMS patients again show no statistically significant difference for this population when compared to rural NCMS patients (Coef = 0.069, *p* > 0.10).

Again, for the other independent variables, only two showed statistical significance: educational level and health status. Those patients in the ‘Middle’ educational category, the highest educational category in our regressions, showed significantly lower monthly outpatient visit utilization (Coef = − 0.388, *p* < 0.05). Self-report status again showed itself to be a good predictor of the level of outpatient utilization with patients reporting ‘good’ and ‘fair’ health being significantly less likely to utilize health services than those patients reporting ‘poor’ health status (Good: Coef = − 0.825, *p* < 0.05; Fair: Coef = − 0.416, *p* < 0.001).

As with the monthly inpatient visit regression, seven of our variables had no significant findings: insurance coverage, age, sex, marital status, employment status, BMI, and per capita consumption.

## Discussion

The NCMS provides health insurance to one of the largest populations in the world with more than 800 million rural Chinese covered; about 98.7% of the total rural population [5]. The system, which was started in 2002, was initially designed primarily to ensure that rural Chinses residents received basic health care services and enable insured citizens to enjoy the equivalent inpatient and outpatient benefit packages for a flat-rate fee. However, as we noted earlier, with China’s expanding economic prosperity a swelling number of rural residents, estimated at 277.5 million by 2015, had migrated from rural to urban areas to take advantage of the expanding employment opportunities. While this migration to urban areas drove China’s economic transformation by supplying essential workers for its economy and the rapid growth of its cities, it also afforded these workers much better job opportunities and accompanying higher incomes.

Many of these rural migrants had NCMS coverage. China’s national policy has a long history based in locality-based activities that depend on the hukou as an organizing principle. NCMS urban residents with a rural hukou designation, as we reported earlier, have migrated from a rural to urban area and we hypothesized this would lead to increased levels of health care resource utilization. In this case, the higher economic profiles of NCMS urban patients enables them to potentially access more healthcare services. While contrasting evidence suggests that there is the possibility that other factors, like the lack of knowledge about NCMS benefits, difficulty dealing with access and different reimbursement policies, and a general absence of good health practices, might ameliorate these potential advantages in the NCMS urban resident designation.

There are other important reasons to be concerned about utilization. With migrant workers employed and living in the city, it was more difficult for them to go back and seek medical care and, consequently, result in lower levels of NCMS utilization. Also, importantly, poor rural counties are much more likely to have limited inpatient and outpatient resources compared to urban areas and very limited resources to support coverage of health care in the more expensive urban hospitals. This has raised the question of whether the cities where migrants move to work should contribute towards insuring against medical costs. Prior research studying the impact of NCMS on migrants showed that 55.2% of migrants in comparison with 24.6% of non-migrants received no reimbursement from the NCMS [14]. This policy was changed but not until the beginning of 2017 when the National Health Planning Commission issued a NCMS cross-provincial settlement reimbursement policy was implemented.

Hence, a robust literature has focused on economic disparities between urban and rural residents and urban and rural NCMS migrants and has found that economic advantages do exist for urban residents [5, 14, 21–25]. For example, Qui, et al. found that while migration may contribute to improving household finances, the new policy was unfair to migrants. The authors noted that although current NCMS policy requires the enrollment unit to be a household, which tends to avoid issues related to adverse selection in the short term, continuing barriers to “portability” will potentially lead to lower participation in the longer term as greater numbers continue to leave rural counties across China.

Consequently, there have been few studies that looked specifically at utilization levels between these two groups of NCMS beneficiaries. Our study was designed to evaluate and report potential disparities in healthcare utilization among urban and rural NCMS patients in 2015 that were all initially registered in a rural hukou in China and part of this massive population shift. Did these potential advantages for NCMS migrants that had moved from a rural to urban areas result in a resource utilization advantage for these migrants over their former rural NCMS neighbors?

Our regression findings on 12,190 urban and rural NCMS patients from the CHARLS survey, controlling for important demographic characteristics of these patients, find no significant difference in inpatient or outpatient utilization for urban and rural NCMS patients. We conclude that in 2015, notwithstanding the potential difficulties for both urban and rural populations in NCMS, there were no apparent disparities in (1) whether individuals received any inpatient or outpatient care during 2015 and (2) for those individuals that did receive care, the extent of the variation in the number of inpatient and outpatient visits among each group.

Our findings also suggest that some of the explanations about the disparities require some nuance. Among rural NCMS beneficiaries, demographics typically reflect that those NCMS beneficiaries migrating to urban areas are younger, better educated, more affluent, and generally have a better self-reported health status than their rural beneficiary counterparts. Our CHARLS NCMS sample in 2015 reflects many of these aspects. We find that NCMS beneficiaries with an urban hukou are younger, have higher levels of education, more like to work in non-agricultural settings, rate their health status higher, and have higher levels of annual income. However, their prevalence of chronic conditions like hypertension and both diabetes and hypertension are higher in urban NCMS residents than their rural NCMS counterparts.

In addition, urban NCMS beneficiaries have a higher percentage in our sample where their BMI shows them to be overweight or obese. Thus, while the financial advantages of NCMS migration may be a benefit for migrants, the prospective stress and consequences of urban life also have a cost. There is a growing disease burden in urban areas in China attributable to nutrition and lifestyle choices and it has become a major public health challenge and troubling new sources of disparities in health-care access for the rural-to-urban migrant population [26].

### Policy recommendation

Currently, the Chinese government is merging the Urban and Rural Medical Scheme (URMS) and NCMS to create a new form health insurance, which is designed to be based on an income-matching monetary method. This balance should help reduce some barriers for rural NCMS migrants to access healthcare services due to their hukou status and offer higher reimbursement rates in both inpatient and outpatient visits. Our results indicate support for the combination of URMS and NCMS [27] especially as these financial increases in payments assist access for the rural hukou residents.

This type of reform might also consider some modifications to have a more effective application and permit greater details about healthcare utilization patterns and priorities. One addition that could improve the incentives associated with using healthcare services is a prospective payment model that could be suited and adopted to China’s needs. This could include a diagnosis-related group (DRG) payment type model for hospitals and risk-adjusted capitation payment method for primary-care providers. A DRG payment system classifies patient services into discrete categories and standardizes prospective payment to hospitals based on these services and encourages cost containment initiatives [28, 29].

There has been a growing interest in using DRGs payment to reimburse inpatient care worldwide. A systematic review of 23 articles representing 13 studies on the effects of DRGs payment on hospital healthcare in China concluded that DRGs payment may slightly improve the efficiency but impair the equity and quality of healthcare, especially for patients not covered by the DRG payment model, and also may result in up-coding of medical records. Furthermore, it was not clear if DRG payments constrain total expenditures or out-of-pocket costs. The answer to the cost question appeared to depend on the components designed in the DRG payment model. The authors concluded that policymakers should very carefully consider each component of DRGs payment design against policy goals and suggested well-designed randomized trials or comparative studies would help strengthen the evidence [30].

Nevertheless, in spite of these important concerns, one important benefit of a well-designed DRG model for China is that the details of the DRG scheme provide critical patient related information that can be used to more extensively evaluate differences and disparities in populations. So, for example, typically DRGs standardize patient classifications associated with an inpatient stay from the time of admission to discharge. The DRG can include any services performed by an outside provider. DRGs categorize patients with respect to diagnosis, treatment and length of hospital stay. The assignment of a DRG is typically designed based some permutation of these patient variables: principal diagnosis, secondary diagnosis (es), surgical procedures performed, comorbidities and complications, patient’s age and sex, and discharge status and this information could be modified. We posit that such data and information from a DRG type system is essential to understanding any potential disparity issues.

Another potential policy opportunity is the development of an effective risk-adjusted payment model. A risk-adjusted capitation payment method is typically designed to compensate a fixed level of payment for each patient per unit of time or service typically paid in advance to the primary providers for their delivered healthcare services. The actual amount paid is typically based on three characteristics of the healthcare encounter: 1) the ranges of services being provided, 2) the number of patients involved, and 3) the time-period during which the services are provided. Capitation rates are normally developed using local costs and average utilization of services and therefore can vary from one region or district of the country to another.

Capitation payments can be designed to control the level or extent of health care resource use by potentially putting the physician or provider at financial risk for their services to patients. Simultaneously, these risk-adjusted protocols need to be closely monitored in order to ensure that patients do not receive suboptimal care through either the under-utilization of health care services by patients or the withholding of health care services by providers. The rates of resource utilization have to be closely monitored and the reports made public to safeguard the public and ensure health care quality. Furthermore, these systems can be linked to financial rewards, such as bonuses or penalties to facilitate or discourage the use of certain utilization practices [31, 32].

Consequently, risk-adjusted payments can incentivize providers to keep costs down to avoid exceeding the fixed reimbursement amount while patients can avoid the travel costs associated with going back to their original registration areas and can obtain health care based on their needs. These two payment approaches have been effective in many other countries and could be advantageous to the Chinese government [33, 34].

### Limitations

There are several study limitations we should note. First, we did not consider the province as a variable. Due to their limited sample size in the NCMS survey, we cannot reasonably classify participants based on their province status. Since NCMS is jointly funded by central and local governments, it may be an important factor to analyze NCMS in different provinces in future studies [35]. Second, because we cannot evaluate the quality of a healthcare visit, we assume the quality across rural and urban hukou residents for inpatient and outpatient visits are similar and that economic differences are not reflections of quality differences in care. Third, we were not able to identify the location where urban and rural beneficiaries receive their inpatient care and outpatient care and, thus, we cannot establish whether repeat medical treatments happened in the same or a different healthcare facility.

Fourth, clearly, moral hazard and adverse selection are two potential factors that might influence our results. That is, a potentially critical problem with voluntary group insurance, like the NCMS model, is that the participants can chose a risky or risk-adverse healthcare strategies and these decisions can result in selection bias among the various subgroups [36]. While we assume these differences are randomly distributed across the sample, we recognize it can have an important impact of healthcare decision making among hukou residents. Fifth, all survey data about health care utilization and living standards were self-reported and may have led to a reporting bias. Finally, CHARLS underrepresents residents under 45 years old and our results may not accurately reflect younger residents.

## Conclusions

Our regression findings on 12,190 urban and rural NCMS patients from the CHARLS survey, controlling for important demographic characteristics of these patients, find no significant difference in inpatient or outpatient utilization for urban and rural NCMS patients. We conclude that in 2015 there were no apparent disparities in (1) whether individuals received any inpatient or outpatient care during 2015 and (2) for those individuals that did receive care, the extent of the variation in the number of inpatient and outpatient visits among each group. These results suggest that utilization concerns about urban and rural disparities in NCMS patients do not appear to be a problem at this point in time.

## Data Availability

The datasets analyzed during the current study are publicly available in the CHARLS repository at http://charls.pku.edu.cn/en. We used the 2015 dataset here.

## Abbreviations

CHARLS: China Health and Retirement Longitudinal Study
DRG: diagnosis-related groups
NCMS: New Cooperative Medical Scheme
SMI: Social Medical Insurance
PSU: primary sampling unit
PPS: probability proportional-to-size
URMS: Urban and Rural Medical Scheme
